# Early Triage of Critically Ill Adult Patients With Mushroom Poisoning: Machine Learning Approach

**DOI:** 10.2196/44666

**Published:** 2023-03-21

**Authors:** Yuxuan Liu, Xiaoguang Lyu, Bo Yang, Zhixiang Fang, Dejun Hu, Lei Shi, Bisheng Wu, Yong Tian, Enli Zhang, YuanChao Yang

**Affiliations:** 1 State Key Laboratory of Information Engineering in Surveying, Mapping and Remote Sensing Wuhan University Wuhan China; 2 Department of Gastroenterology Renmin Hospital of Wuhan University Wuhan China; 3 Department of Internal Medicine Renmin Hospital of Xianfeng Enshi China; 4 Department of Nephrology Minda Hospital of Hubei Minzu University Enshi China; 5 Department of General Surgery Renmin Hospital of Xianfeng Enshi China; 6 Department of Internal Medicine Renmin Hospital of Laifeng Enshi China; 7 Department of General Surgery Central Hospital of Hefeng Enshi China; 8 Department of Gastroenterology Renmin Hospital of Xuanen Enshi China

**Keywords:** mushroom poisoning, triage, model, machine learning, XGBoost, extreme gradient boosting

## Abstract

**Background:**

Early triage of patients with mushroom poisoning is essential for administering precise treatment and reducing mortality. To our knowledge, there has been no established method to triage patients with mushroom poisoning based on clinical data.

**Objective:**

The purpose of this work was to construct a triage system to identify patients with mushroom poisoning based on clinical indicators using several machine learning approaches and to assess the prediction accuracy of these strategies.

**Methods:**

In all, 567 patients were collected from 5 primary care hospitals and facilities in Enshi, Hubei Province, China, and divided into 2 groups; 322 patients from 2 hospitals were used as the training cohort, and 245 patients from 3 hospitals were used as the test cohort. Four machine learning algorithms were used to construct the triage model for patients with mushroom poisoning. Performance was assessed using the area under the receiver operating characteristic curve (AUC), decision curve, sensitivity, specificity, and other representative statistics. Feature contributions were evaluated using Shapley additive explanations.

**Results:**

Among several machine learning algorithms, extreme gradient boosting (XGBoost) showed the best discriminative ability in 5-fold cross-validation (AUC=0.83, 95% CI 0.77-0.90) and the test set (AUC=0.90, 95% CI 0.83-0.96). In the test set, the XGBoost model had a sensitivity of 0.93 (95% CI 0.81-0.99) and a specificity of 0.79 (95% CI 0.73-0.85), whereas the physicians’ assessment had a sensitivity of 0.86 (95% CI 0.72-0.95) and a specificity of 0.66 (95% CI 0.59-0.73).

**Conclusions:**

The 14-factor XGBoost model for the early triage of mushroom poisoning can rapidly and accurately identify critically ill patients and will possibly serve as an important basis for the selection of treatment options and referral of patients, potentially reducing patient mortality and improving clinical outcomes.

## Introduction

Approximately 5-10 per 100,000 people die annually from accidental wild mushroom poisoning worldwide, mainly in European countries, the United States, Japan, China, and Iran [1,2]. The American Association of Poison Control Centers reported 86,462 (10,808 cases/year) cases of mushroom exposure from 2012 to 2019. In Japan, there were 1920 cases of mushroom poisoning from 2001-2010, with a morbidity and mortality rate of 0.52% [3]. China’s foodborne disease outbreak surveillance system recorded 10,036 outbreaks of mushroom poisoning between 2010 and 2020, resulting in 38,676 illnesses, 21,967 hospitalizations, and 779 deaths [4]. According to the National Health and Family Planning Commission and the Chinese Center for Disease Control and Prevention, mushroom poisoning is the leading cause of death from food poisoning in China [5,6]. Rapid and effective triage is essential for the early treatment of patients with mushroom poisoning and the effective allocation of hospital resources.

The HOPE6 and TALK scores [7] are recommended by the Emergency Physicians Branch of the Chinese Physicians Association, the Chinese Emergency Medical Specialists Consortium, the Emergency Resuscitation and Disaster Medicine Specialty Committee of the Chinese Physicians Association, and the Beijing Emergency Medicine Society, and they serve as an important basis for patient treatment plan determination and are the most widely used condition assessment models in clinical work in China. However, there are some limitations of the abovementioned condition assessment methods: (1) Medical technology is limited in areas with a high incidence of mushroom poisoning, and toxin detection tools and some treatment tools are not available in primary hospitals. (2) Some of the assessment items are poorly clinically operable in practice; for example, patients basically cannot provide the exact information of the poisonous mushroom consumed to determine the type of mushroom. (3) The model may be overly simplistic for complex clinical events such as the development of a critical illness because it assumes risk is a linear mix of numerous factors. (4) The scoring model is proposed based on the literature rather than clinical data, the existing mushroom poisoning literature is mostly summarized based on severe cases, and the patient’s condition may be greatly overestimated [8].

In recent years, with the emergence of biomedical big data, machine learning has attracted great attention for developing clinical informatics tools for disease diagnosis, staging, and prognosis [9-11] and has been used in personalized medicine [12-15]; therapeutics [16,17]; surgery [18,19]; radiology [20-25]; and hematology, oncology, and pathology [14,26-29]. It has been demonstrated that machine learning may predict clinical outcomes more accurately than traditional statistical models, particularly when applied to huge data sets [30]. Machine learning algorithms, in contrast to regression-based methods, are capable of capturing higher-order nonlinear interactions among predictors [31]. In this paper, we present the first machine learning–based early condition assessment model for mushroom poisoning assessment, which aims to improve the efficiency and accuracy of condition assessment.

## Methods

**Figure 1:**
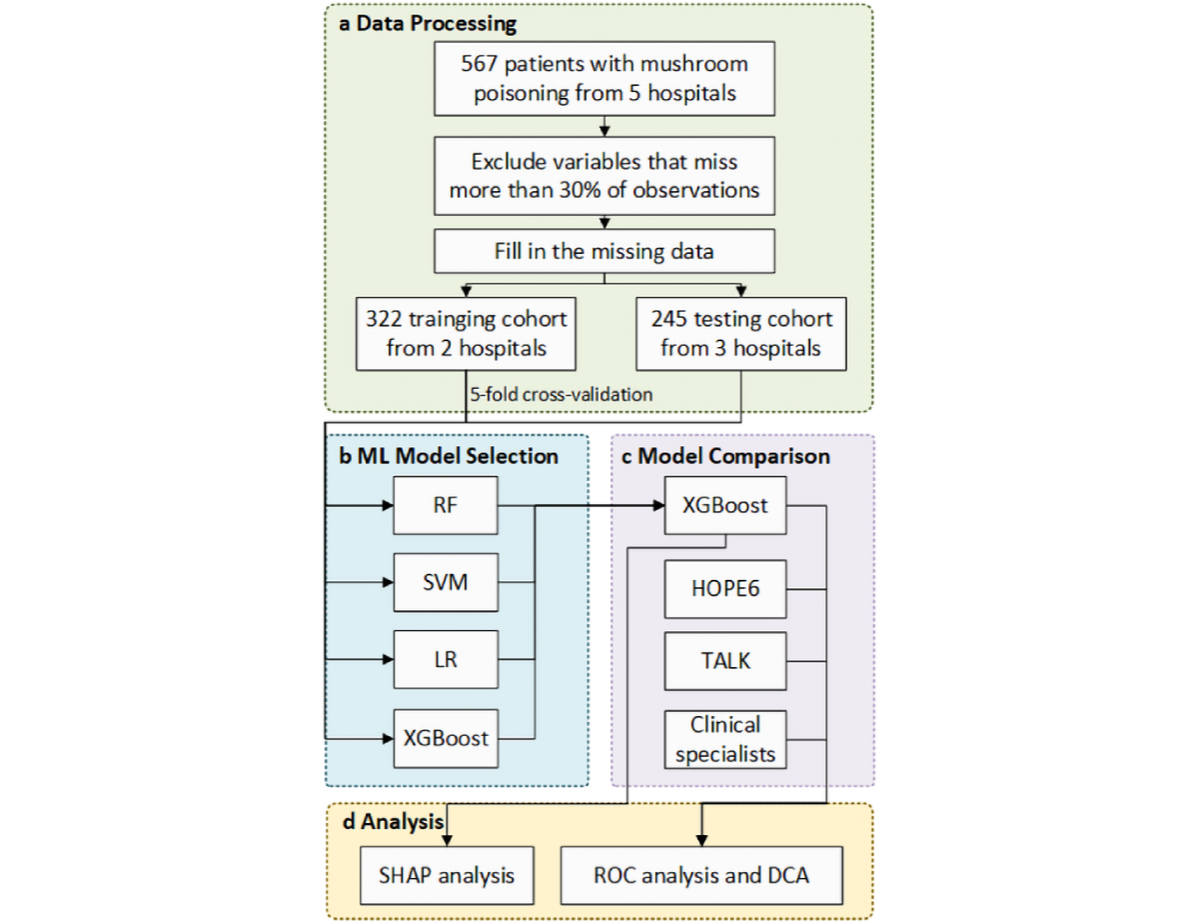
Study flowchart. The phases of data processing, model selection, model comparison, and analysis are illustrated. DCA: decision curve analysis; LR: logistic regression; ML: machine learning; RF: random forest; ROC: receiver operating characteristic; SHAP: Shapley additive explanations; SVM: support vector machine; XGBoost: extreme gradient boosting.

Our study was divided into 4 steps, and the flowchart of the whole work is shown in Figure 1.

### Patient Population

We collected data on patients with wild mushroom poisoning admitted to 5 county hospitals in Enshi, Hubei Province, China, between January 2010 and May 2022. Critical illness was defined as the occurrence of an admission to an intensive care unit, hemodialysis therapy, referral to a higher-level hospital, or death. We collected 24-hour data from 567 patients from 5 hospitals. We used data from 322 patients from 2 of the hospitals as the training set, of which 56 were critically ill and 245 were noncritically ill. Data from 245 patients from 3 other hospitals were used as the test set to assess the performance of our model, of which 43 were critically ill and 202 were noncritically ill.

We used the following inclusion criteria to develop the condition assessment model: (1) patients older than 14 years of age and (2) definite consumption of wild mushrooms before the onset of the disease. Data used in the diagnostic model were excluded when the following conditions were met: (1) age younger than 14 years; (2) possible concurrent consumption of other foods causing acute poisoning; and (3) previous cardiac, hepatic, and renal disorders, as well as mental disorders.

### Data Preprocessing

To ensure the reliability of the results and that model use characteristics were readily available, we excluded variables that omitted more than 30% of the observations. The missing values of continuous attributes were filled with the mean value of each attribute, and the missing values of discrete attributes were filled with the mode of each attribute. The values of each feature were normalized for the support vector machine and logistic regression.

### Development of Machine Learning Model

Four popular machine learning classification algorithms, including extreme gradient boosting (XGBoost) [32], random forest, support vector machine, and logistic regression, were applied in this study to build the classification models. We implemented machine learning algorithms using Python (version 3.9; Python Software Foundation) and several Python modules (*panda*, *numpy*, *scipy*, *sklearn*, *xgboost*, *shap*, and *matplotlib*). Hyperparameter tuning was performed by a grid search based on 5-fold cross-validation to select the best area under the receiver operating characteristic curve (AUC) value for the classification models.

A proper model interpretation must be supplied for the machine learning model. Model predictions were interpreted using Shapley additive explanations (SHAP) [33,34]. SHAP is a model-independent interpretation technique that helps to interpret the results of prediction models. The interpretation is based on the SHA*P* value for each feature, which indicates the feature’s contribution to the risk of being predicted as critically ill. Having a positive SHA*P* value indicates that the corresponding feature contributes to a higher risk of the patient being critically ill and is a risk factor. On the other hand, having a negative SHA*P* value indicates that the corresponding feature contributes to a lower risk of the patient being critically ill and is a protective factor.

### Statistical Analysis

To validate the performance of the model, we compared the best machine learning model with the HOPE6 and TALK scoring models [7]. In addition, we included the results of each patient’s primary care physician’s assessment of the patient’s condition to explore the diagnostic performance of our model compared with that of the physician’s judgment.

We evaluated model performance in the test set by calculating (1) the AUC; (2) decision curve analysis (DCA); and (3) sensitivity, specificity, positive predictive value, negative predictive value, positive likelihood ratio, and negative likelihood ratio. The AUC is often used to assess the performance of various prediction models and is robust to category imbalance [35]. Based on the receiver operating characteristic curve, we chose the best predictive value (i.e., the value that is closest to the perfect model) for cases to fix the category imbalance for whether the condition is critical or not [36]. By calculating the AUC of different models, the discriminatory ability of different models can be compared. However, the AUC only focuses on the overall accuracy of the models and does not focus on the relationship between benefit and risk associated with different cutoff values in different models. DCA, on the other hand, permits the assessment of the range of threshold probabilities for a model to have value, the magnitude of the benefit, and the best model among numerous candidates. DCA figures out the “clinical net benefit” of one or more predictive models over a range of threshold probabilities. A threshold probability is a minimum chance that a disease needs further intervention, and the “clinical net benefit” takes into account the relative harms of false positives and false negatives [37].

### Ethics Approval

This study was approved by the Ethical Committee of Renmin Hospital of Xianfeng, and informed consent was waived because this study was retrospective and used deidentified data (XFRY2021-12). The privacy and confidentiality of all individuals included in this study were strictly protected, and their data were used only for the purposes of this research.

## Results

### Patient Characteristics

The patient cohort participating in this study included data from 567 cases of mushroom poisoning of patients admitted to 5 county hospitals in the Enshi area. The case data included the following types of mushroom poisoning: gastroenteritis, neuropsychiatric symptoms, acute liver damage, acute renal failure, myocardial injury, and combined types [7]. For all data, the length of stay was 4.45 (95% CI, 3.45-5.44) and 3.52 (95% CI 3.21-3.82) days for critically ill and noncritically ill patients, respectively, and the cost of hospitalization was ¥11,113.09 (95% CI ¥8059.92-¥14,166.27; ¥1=US $0.14) and ¥2392.08 (95% CI ¥2188.34-¥2595.82), respectively.

Table 1 shows the baseline data of patients. The majority of the indicators did not differ significantly between the training set and the test set, and the remaining indicators (*P*<.01) were excluded from the machine learning model.

**Table 1:** Patient baseline characteristics.

Variables		Training set (n=322)	Test set (n=245)	*P* value
Age (years), mean (SD)		50.89 (17.37)	55.62 (15.9)	<.001
**Sex, n (%)**				.21
	Male	155 (48.1)	131 (53.5)	
	Female	167 (51.9)	114 (46.5)	
**Critical illness, n (%)**				.96
	Yes	56 (17.4)	43 (17.6)	
	No	266 (82.6)	202 (82.4)	
**Vital signs, mean (SD)**				
	Systolic pressure	120.9 (20.1)	122.43 (21.45)	.39
	Diastolic pressure	76.07 (11.83)	77.82 (13.09)	.10
	Respiratory rate	18.89 (2.4)	19.74 (1.86)	<.001
	Temperature	36.5 (0.4)	36.4 (0.43)	<.001
	Heart rate	81.39 (13.87)	79.58 (14.29)	.13
**Blood routine test, mean (SD)**				
	WBC^a^	11.98 (39.69)	9.39 (4.18)	.31
	RBC^b^	4.63 (0.79)	4.62 (4.18)	.89
	Hemoglobin	137.57 (26.6)	137.74 (24.52)	.94
	Hematocrit	42.88 (8.74)	37.87 (13.71)	<.001
	Heart rate	81.39 (13.87)	79.58 (14.29)	.13
**Liver function, mean (SD)**				
	TBiL^c^	16.89 (11.65)	17.7 (15.98)	.49
	DBiL^d^	6.05 (5.73)	6.09 (8.27)	.94
	IBiL^e^	10.4 (5.64)	12.23 (12.14)	.02
	ALT^f^	106.23 (380.7)	142.23 (689.61)	.43
	AST^g^	152.04 (713.41)	166.09 (824.13)	.83
**Kidney function, mean (SD)**				
	Urea	7.03 (3.17)	7.69 (4.5)	.08
	Creatinine	91.27 (54.42)	100.15 (94.5)	.16
**Coagulation function, mean (SD)**				
	PT^h^	13.14 (3.52)	12.88 (4.86)	.48
	TT^i^	13.82 (2.57)	15.21 (16.76)	.17
	PPT^j^	31.65 (6.24)	33.88 (15.91)	.03
	Fibrinogen	2.77 (0.76)	3.01 (0.83)	<.001
	INR^k^	1.08 (0.34)	1.06 (0.3)	.39
**Serum electrolytes, mean (SD)**				
	Serum potassium	4.94 (17.39)	4.97 (14.5)	.99
	Serum sodium	138.24 (11.62)	136.21 (16.4)	.09
**Other blood biochemistry and enzymatic parameters, mean (SD)**				
	CO2CP^l^	22.6 (9.81)	23.59 (13.71)	.15
	CK^m^	394 (1981.36)	49.29 (527.65)	.16
	CK-MB^n^	38.22 (138.74)	35.89 (118.64)	.84
	LDH^o^	298.95 (570.85)	342.67 (997.28)	.52

^a^WBC: white blood cell.

^b^RBC: red blood cell.

^c^TBiL: total bilirubin.

^d^DBiL: direct bilirubin.

^e^IBiL: indirect bilirubin.

^f^ALT: alanine transaminase.

^g^AST: aspartate aminotransferase.

^h^PT: prothrombin time.

^i^TT: thrombin time.

^j^PPT: partial thromboplastin time.

^k^INR: international normalized ratio.

^l^CO2CP: carbon dioxide combining power.

^m^CK: creatine kinase.

^n^CK-MB: creatine kinase-MB.

^o^LDH: lactate dehydrogenase.

### Comparison Among the Machine Learning Algorithms

**Figure 2:**
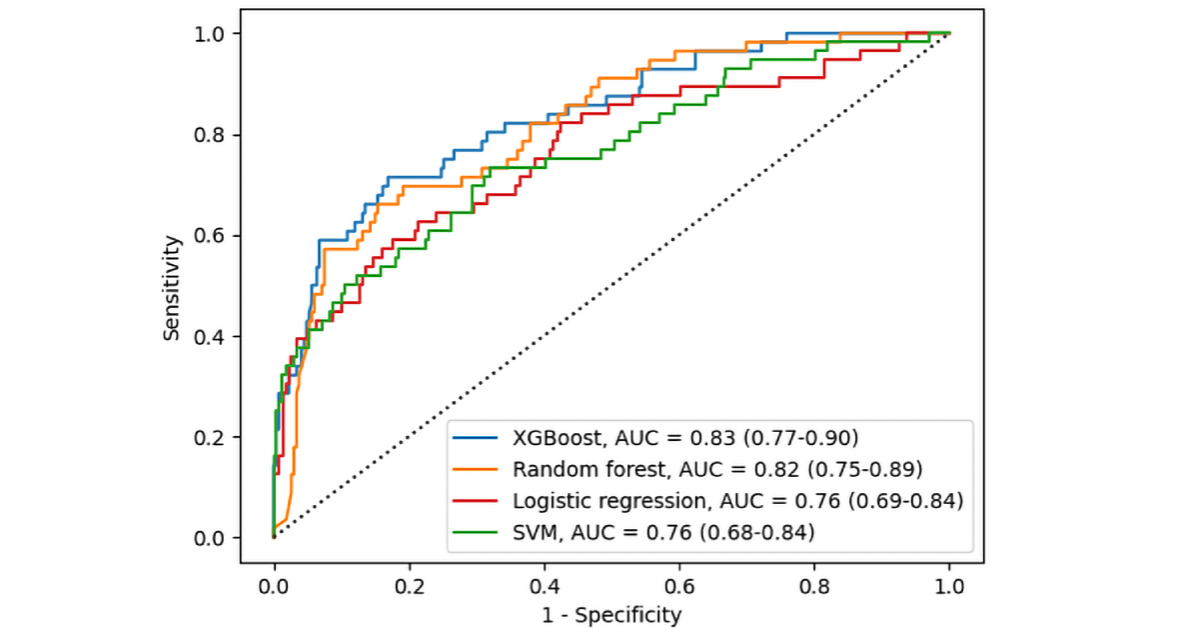
ROC curves for each machine learning algorithm in the 5-fold stratified cross-validation. Diagonal dotted lines represent the random classifier. AUC: area under the receiver operating characteristic curve; ROC: receiver operating characteristic; SVM: support vector machine; XGBoost: extreme gradient boosting.

**Figure 3:**
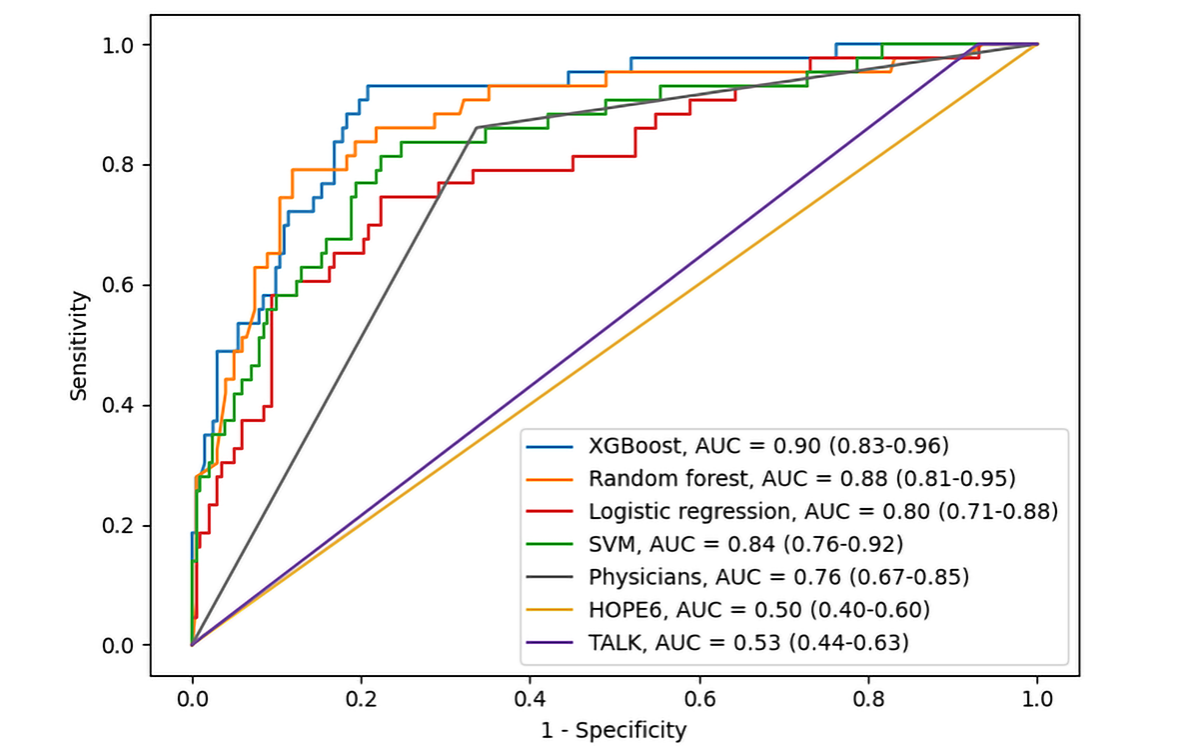
ROC curves for machine learning algorithms, HOPE6, TALK, and physicians’ assessment in the test set. AUC: area under the receiver operating characteristic curve; ROC: receiver operating characteristic; SVM: support vector machine; XGBoost: extreme gradient boosting.

**Figure 4:**
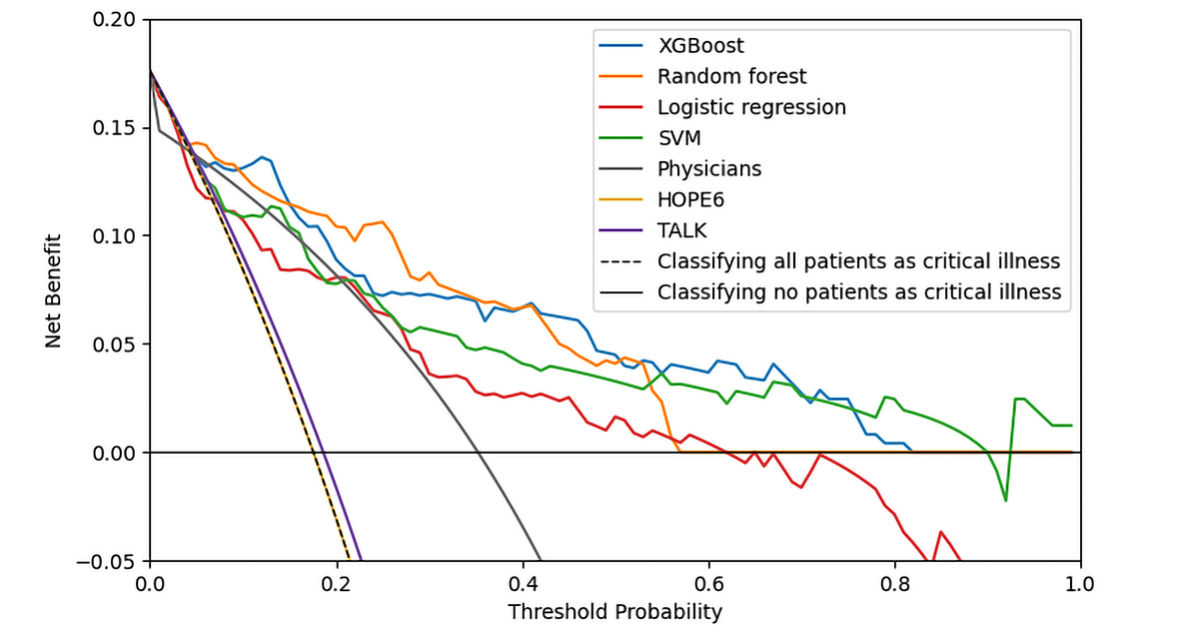
Decision curve analysis on the test set. The x-axis represents the threshold probability of the hospitalization outcome. The y-axis represents the net benefit. The curves (decision curves) represent the threshold probabilities of net benefit outcomes for the 6 models (4 machine learning models, HOPE6 model, and TALK model), physician classification, and the 2 clinical alternatives (classifying no patients as critical vs classifying all patients as critical) within the specified range. SVM: support vector machine; XGBoost: extreme gradient boosting.

Among the algorithms, XGBoost achieved the highest AUC value, with AUCs of 0.83 (95% CI 0.77-0.90) and 0.90 (95% CI 0.83-0.96) in the internal and external validation sets (Figures 2 and 3). In the DCA (Figure 4), the XGBoost model had a greater net benefit compared to other methods over a wide range of threshold probabilities. As a result, we selected XGBoost as a suitable algorithm for developing the prediction model and conducted additional analyses to determine its predictive validity. 

**Figure 5:**
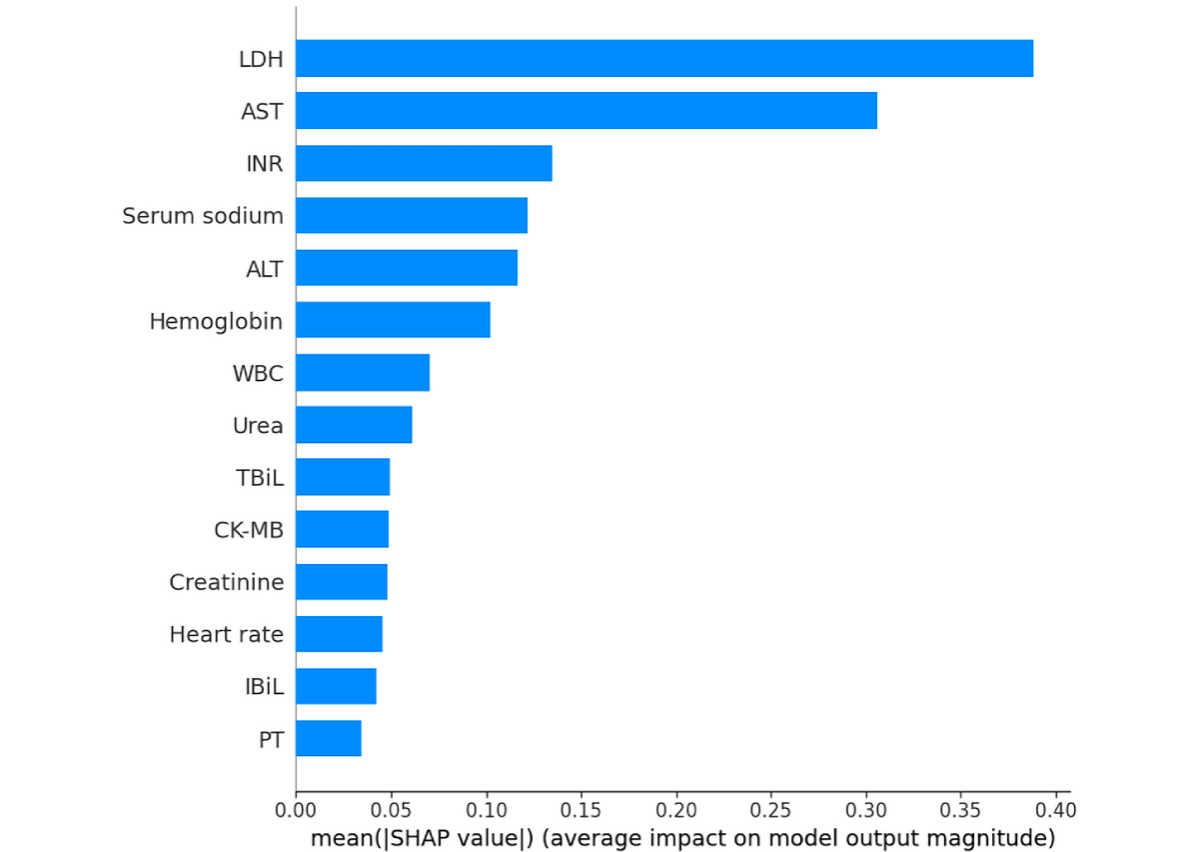
Ranking of feature importance indicated by SHAP. ALT: alanine transaminase; AST: aspartate aminotransferase; CK-MB: creatine kinase-MB; IBiL: indirect bilirubin; INR: international normalized ratio; LDH: lactate dehydrogenase; PT: prothrombin time; SHAP: Shapley additive explanations; TBiL: total bilirubin; WBC: white blood cell.

**Figure 6:**
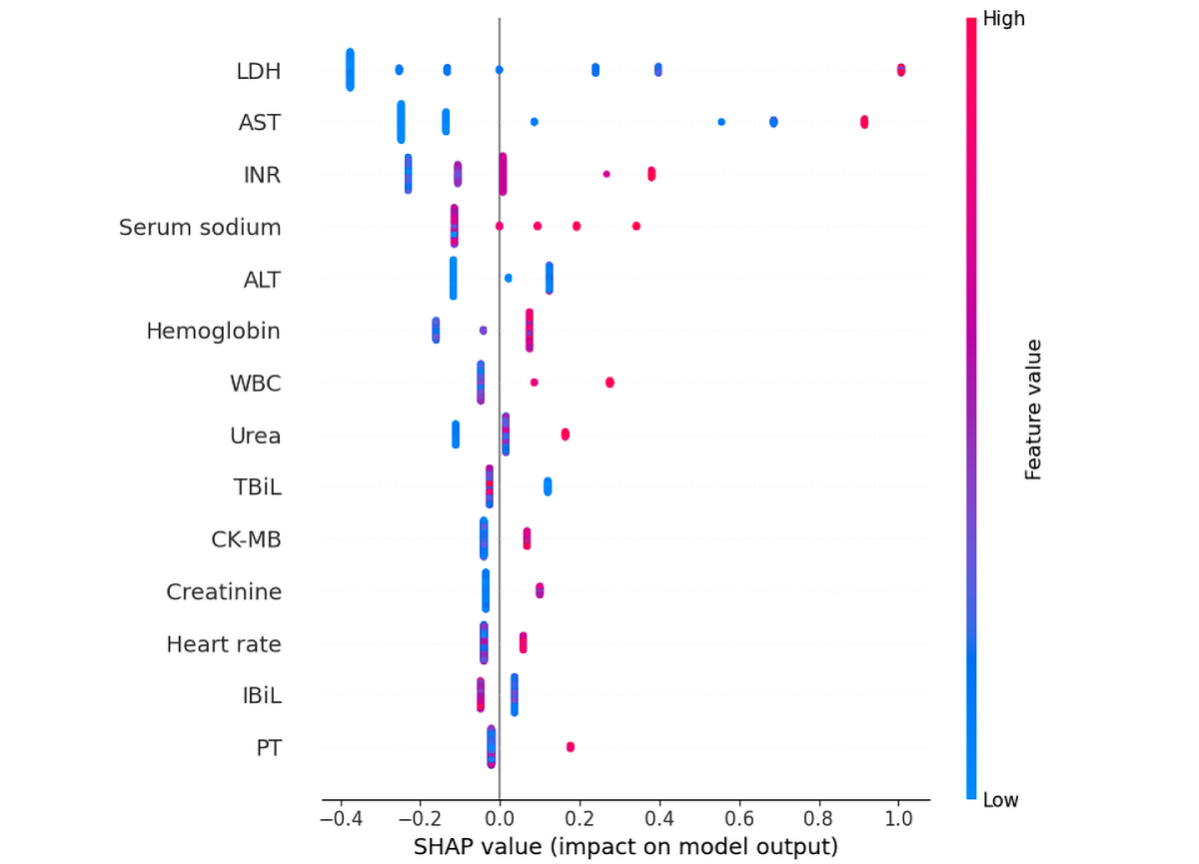
Attributes of the model's features. Each line is a feature, and the horizontal coordinate is the SHA*P* value, which shows how much that feature affected the outcome. Every point on the graph is a sample. The value of a feature goes up as the color gets redder, and it goes down as the color gets bluer. ALT: alanine transaminase; AST: aspartate aminotransferase; CK-MB: creatine kinase-MB; IBiL: indirect bilirubin; INR: international normalized ratio; LDH: lactate dehydrogenase; PT: prothrombin time; SHAP: Shapley additive explanations; TBiL: total bilirubin; WBC: white blood cell.

The feature ranking interpretation of the XGBoost model based on the SHAP algorithm (Figure 5) shows that lactate dehydrogenase (LDH), aspartate aminotransferase (AST), international normalized ratio (INR), serum sodium, alanine transaminase (ALT), hemoglobin, white blood cell, urea, total bilirubin (TBiL), creatine kinase-MB (CK-MB), creatinine, heart rate, indirect bilirubin (IBiL), and prothrombin time (PT) were important features of the XGBoost model. LDH and AST were the most influential factors, and their contribution was considerably greater than that of other indicators (Figure 5). Overall, the characteristics of LDH, AST, INR, serum sodium, ALT, hemoglobin, white blood cell, urea, CK-MB, creatinine, heart rate, and PT were positively correlated with the results and were risk factors; meanwhile, TBiL and IBiL were negatively correlated with the results and were protective factors (Figure 6).

### Comparison of the Performances of the XGBoost, HOPE6, TALK, and Physicians’ Assessment

In terms of the AUC, all the machine learning models performed significantly better than HOPE6, TALK, and physicians’ assessment (Figure 3). Moreover, across a wide range of threshold probabilities (or clinical preferences), all machine learning models performed better than HOPE6, TALK, and physicians’ assessment (Figure 4). In terms of AUC and DCA, XGBoost was the top model (Figures 3 and 4).

The sensitivity, specificity, positive predictive value, negative predictive value, positive likelihood ratio, and negative likelihood ratio of each model are shown in Table 2. Almost all patients were classified as critically ill in the HOPE6 and TALK scores (specificity: 0.00, 95% CI 0.00-0.02 for HOPE6 and 0.07, 95% CI 0.04-0.11 for TALK). In the test set, the XGBoost model had a sensitivity of 0.93 (95% CI 0.81-0.99) and a specificity of 0.79 (95% CI 0.73-0.85), whereas the physicians’ assessment had a sensitivity of 0.86 (95% CI 0.72-0.95) and a specificity of 0.66 (95% CI 0.59-0.73). The results indicate that the current diagnostic model can more precisely evaluate a patient’s condition, assist physicians in formulating more precise treatment plans, lessen the harm caused by incorrect treatment, and reduce patients’ treatment expenses.

**Table 2:** Comparison of the prediction ability of the extreme gradient boost (XGBoost model), HOPE6, TALK, and physicians’ assessment on the test set.

	Sensitivity (95% CI)	Specificity (95% CI)	PPV^a^ (95% CI)	NPV^b^ (95% CI)	PLR^c^ (95% CI)	NLR^d^ (95% CI)
XGBoost	0.93 (0.81-0.99)	0.79 (0.73-0.85)	0.49 (0.38-0.60)	0.98 (0.95-1.00)	4.47 (3.38-5.93)	0.09 (0.03-0.26)
HOPE6	1.00 (0.92-1.00)	0.00 (0.00-0.02)	0.18 (0.13-0.23)	—^e^	1.00 (1.00-1.00)	—
TALK	1.00 (0.92-1.00)	0.07 (0.04-0.11)	0.19 (0.14-0.24)	1.00 (0.77-1.00)	1.07 (1.03-1.12)	—
Physicians’ assessment	0.86 (0.72-0.95)	0.66 (0.59-0.73)	0.35 (0.26-0.45)	0.96 (0.91-0.98)	2.56 (2.04-3.21)	0.21 (0.10-0.44)

^a^PPV: positive predictive value.

^b^NPV: negative predictive value.

^c^PLR: positive likelihood ratio.

^d^NLR: negative likelihood ratio.

^e^Not available.

## Discussion

### Principal Findings

Mushroom poisoning is a global food safety event. Early triage of patients with mushroom poisoning is essential for the formulation of treatment options and reduction of mortality. The objective of this study was to develop a machine learning–based triage model that could assess whether a patient with mushroom poisoning was critically ill within 24 hours of admission to support clinical decision-making. To our knowledge, this is the first time a machine learning algorithm has been used for the early triage of mushroom poisoning. The research demonstrates that the model developed using the XGBoost algorithm is superior to previous methods for triaging critically ill patients with mushroom poisoning. In addition, we discovered that liver dysfunction had the greatest impact on the model (more than 50%), with LDH and AST being the 2 most influential factors.

### Comparison With Prior Work

In this paper, we compared 4 machine learning models, 2 scoring models (HOPE6 and TALK), and clinical experts’ assessment results and found that machine learning models outperformed conventional methods. First, machine learning algorithms are entirely data driven, whereas scoring models and physicians’ evaluations are based on expert knowledge. Second, machine learning algorithms can learn and infer nonlinear higher-order connections between clinical factors and patient outcomes. Scoring models have the advantage of being simple to calculate and interpret; however, for complex clinical episodes (e.g., progression to critical illness), they may be overly simplistic in assuming that the severity of a patient’s condition is a linear combination of multiple factors.

XGBoost, a cutting-edge tree-based gradient boosting method, allowed us to create more accurate predictive models than other machine learning models; consequently, it was selected as our final model. The XGBoost model had greater sensitivity (0.93, 95% CI 0.81-0.99) and specificity (0.79, 95% CI 0.73-0.85) than physicians’ assessment (sensitivity: 0.86, 95% CI 0.72-0.95; and specificity: 0.66, 95% CI 0.59-0.73). As expert consensus, the HOPE6 and TALK models have not been validated by clinical data, and because the identification and treatment of critically ill patients have important clinical implications, scoring models based on expert experience will overestimate the condition of every patient. In this study, the HOPE6 and TALK models have extremely high sensitivity (1.00, 95% CI 0.92-1.00) and extremely low specificity (HOPE6: 0.00, 95% CI 0.00-0.02; and TALK: 0.07, 95% CI 0.04-0.11). Consequently, they are able to identify all critical cases and play an important role in clinical practice, allowing critical patients to be treated and the mortality rate to be as low as possible. However, an excessive number of noncritically ill patients will be misidentified as critically ill patients, which may result in the waste of medical resources and physical harm to patients as a result of overtreatment. Therefore, our model has greater advantages for the identification of critically ill patients and may aid physicians in making assessments. Moreover, the model’s ability to adjust cutoff values provides greater flexibility and more nuanced insights into the patient’s condition, making it a useful tool for physicians. The application software created by our current model is available on GitHub [38].

The study identified 14 clinical variables, of which AST, ALT, LDH, INR, PT, TBiL, IBiL, urea, creatinine, and CK-MB were consistent with current clinical evidence. These factors were classified as hepatic (AST, ALT [39-41], LDH [42], TBiL [39], and IBiL [43]), coagulation (INR [39,41] and PT [43]), renal (urea [43] and creatinine[40,44]), and cardiac impairment (CK-MB [42]). Using the SHAP method, we found that the indicators characterizing liver function impairment contributed 57.5% to the prediction results of the model and had the greatest impact. Interestingly, the study also identified several clinical indicators that lack relevant clinical evidence, such as serum sodium, hemoglobin, and heart rate, highlighting the potential for machine learning to identify novel relationships between clinical factors and patient outcomes.

### Limitations

Despite the promising results, the study has several limitations. The machine learning method is data driven, and the model’s performance is dependent on the quality and completeness of the data. Additionally, the study cohort was from a single location in China, limiting the model’s applicability to other regions. Future studies should collect data from more locations to enhance the model’s robustness and generalizability.

### Conclusions

Using routinely accessible data at the time of triage, we found that machine learning has good predictive performance in the triage of patients with mushroom poisoning. Compared with other machine learning algorithms, clinical guidelines, and clinician assessment, the XGBoost model has better diagnostic performance and can be used to select core indicators for a triage model. Machine learning may be a powerful prognostic indicator for early warning in critically ill patients, which has a significant impact on the triage of patients, formulation of treatment options, and the allocation of medical resources.

## Abbreviations

ALT: alanine transaminase
AST: aspartate aminotransferase
AUC: area under the receiver operating characteristic curve
CK-MB: creatine kinase-MB
DCA: decision curve analysis
IBiL: indirect bilirubin
INR: international normalized ratio
LDH: lactate dehydrogenase
PT: prothrombin time
SHAP: Shapley additive explanations
TBiL: total bilirubin
XGBoost: extreme gradient boosting

## Data Availability

The data that support the findings of this study are available from the authors upon reasonable request but cannot be shared with the public due to privacy concerns and restrictions imposed by the institutional review board. The data are available by contacting correspondent authors for collaboration or other reasonable requests with permission from the project directors.
